# Mercurialism in Lying-in Women Undergoing Sublimate Irrigation

**Published:** 1887-08

**Authors:** 


### ON MERCURIALISM IN LYING-IN WOMEN UNDERGOING SUBLIMATE IRRIGATION.

DAKIN makes a valuable contribution to the question of mercurialism following the use of corrosive sublimate as an antiseptic. As is now well known, Tarnier, in 1882, was the first to use this drug as an antiseptic in puerperal cases. The general outcome of its use has been a great diminution in the mortality and morbility of maternities, a result somewhat clouded by occasional cases of poisoning with even fatal results, as many as eight deaths having been reported. The cases known are as follows:

1. *Stadtfeldt's Case* One intra uterine injection, 1:1500, five days after labor. Death on eleventh day. Kidney and intestinal changes found.

2. *Somer's Case.* Vaginal, 1:1000.

3. *Winter's Case.* Intra-uterine douche, 1:1000; vaginal afterward of 1:1000. Death on fourth day. Post-mortem, gangrenous colitis and peritoneal exudation.

4. *Vohtz.* Intra-uterine injection, 1:750, after abortion. Death on eleventh day after intestinal and kidney symptoms.

5. *Partridge.* Two intra-uterine injections, 1:2000; dysenteric symptoms and death.

6 and 7. *Thorn.* Intra-uterine douche after missed abortion, 1:1000; vaginal douche, 1:1000 in forceps case. Renal and internal lesions.

8. *Ziegenspeck.* Death on thirteenth day after three injections of 1:5000. No bad symptoms until second intra-uterine douche on eighth day; patient died five days afterward.

9. *Netzel.* Intra-uterine douche, 1:1500, on seventh day; abdominal pain then complained of; renal symptoms with albumen and lime and lime salts in urine. Death on twenty-second day.

10. *Gustav Braun*. Douche during first stage; episiotomy performed during second stage, and intra-uterine irrigation, 1:1000, after expulsion of placenta. Diarrhœa on third, and collapse on seventh.

11. *Hofmeier*. One douche of 1:1000. Death on sixth day.

In all cases of douching Keller found mercury in urine, and also albumen.

Dakin then gives the results of the use of sublimate irrigation in 170 patients confined in the General Lying-in Hospital, London, under the charge of Dr. William P. Champneys. The sublimate is used as follows: Hands and instruments are disinfected with 1:1000, the vagina is douched after labor with 1:2000, two pints, at a temperature of 115°–120° F., being used. For the first two days three pints of 1:2000 at 110° F. are employed, and then the strength is reduced to 1:4000, The patient occupies the dorsal posture, and after the douche is finished turns on her hands and knees, to evacuate any residuum.

Symptoms of poisoning usually appeared between the fourth and seventh days, and were usually diarrhœa, with blood and tenesmus, sometimes vomiting, patchy tongue, thirst, and loss of appetite, fetor of breath, salivation, with tenderness of teeth and gums, as well as red line on gums. The urine contained albumen in 11 of the cases.

Details of the one fatal case are given, with examination of kidneys.

He comes to the conclusion that a strength of 1:4000 is sufficient for douching.—*Quarterly Compendium of Medical Science.*

